# Effects of COVID-19 pandemic lockdown on the metabolic control of type 2 diabetes mellitus in patients

**DOI:** 10.20945/2359-3997000000621

**Published:** 2023-05-10

**Authors:** Mehmet Ali Eren, Ataman Gönel, Hüseyin Karaaslan, Nida Uyar, Çiğdem Cindoğlu, Tevfik Sabuncu

**Affiliations:** 1 Harran University School of Medicine Department of Endocrinology Sanliurfa Turkey Harran University, School of Medicine, Department of Endocrinology, Sanliurfa, Turkey; 2 Harran University School of Medicine Department of Clinical Biochemistry Sanliurfa Turkey Harran University, School of Medicine, Department of Clinical Biochemistry, Sanliurfa, Turkey; 3 Harran University School of Medicine Department of Internal Medicine Sanliurfa Turkey Harran University, School of Medicine, Department of Internal Medicine, Sanliurfa, Turkey

**Keywords:** COVID-19, pandemic, lockdown, diabetes mellitus, glycated hemoglobin

## Abstract

**Objective:**

The effects of the COVID-19 pandemic on the control of diabetes mellitus in patients are largely unknown. In this study we aimed to analyze the impact of the pandemic and the ensuing lockdown on the management of type 2 diabetes mellitus.

**Subjects and methods:**

A total of 7,321patients with type 2 diabetes mellitus (4,501 from the pre-pandemic period, 2,820 from the post-pandemic period) were studied retrospectively.

**Results:**

The admission of patients with diabetes melitus (DM) decreased significantly during the pandemic (4,501 pre-pandemic vs. 2,820 post-pandemic; p < 0.001). The mean age of patients was statistically lower (51.5 ± 14.0 vs. 49.7 ± 14.5 years; p < 0.001), and the mean glycated hemoglobin (A1c) level was significantly higher (7.9% ± 2.4% vs. 7.3% ± 1.7%; p < 0.001) in the post-pandemic period than in the pre-pandemic. The female/male ratio was similar in both periods (59.9%/40.1% for pre-pandemic, 58.6%/41.4% for post-pandemic; p = 0.304). As calculated by month the pre-pandemic rate of women was higher only in January (53.1% vs. 60.6%, p = 0.02). Mean A1c levels were higher in the postpandemic period than in the same month of the previous year, excluding July and October (p = 0.001 for November, p < 0.001 for others). Postpandemic patients admitted to the outpatient clinic were significantly younger than prepandemic visits for July (p = 0.001), August (p < 0.001) and December (p < 0.001).

**Conclusion:**

The lockdown had detrimental effects on blood sugar management in patients with DM. Hence, diet and exercise programs should be adapted to home conditions, and social and psychological support should be provided to patients with DM.

## INTRODUCTION

COVID-19 is an acute respiratory disease caused by the novel coronavirus. The World Health Organization (WHO)has designated it the been named “Severe Acute Respiratory Syndrome Coronavirus 2” (SARS-CoV-2) by the World Health Organization (WHO) ( 1 , 2 ). In December 2019, it was first diagnosed in China and subsequently spread to nearly every country in the world. On March 11, 2020, the WHO declared the COVID-19 outbreak to be a pandemic ( 3 ). Soon after, several countries started to apply lockdown procedures ( 4 ). Under the pandemic lockdown, to minimize visits to hospitals, patients with chronic diseases were allowed to continue their medications without a doctor's prescription ( 4 ).

Diabetes mellitus (DM) is a chronic metabolic disorder that has become a rapidly growing problem globally, with implications for social life, well-being, and the economic condition of patients ( 5 ). Moreover, DM is one of the most prevalent comorbidities among COVID-19 patients requiring hospitalization ( 6 ). As many other health services were shut down during the pandemic, there was a decrease in access to adequate medical help and hospitalization for patients with non-COVID-19 pathologies ( 7 ). Due to the lockdown, there have been reports of several problems in the management of chronic diseases such as DM.

In light of this information, we aim to analyze the effects of the lockdown on the control of type 2 DM in patients during the pandemic.

## SUBJECTS AND METHODS

We scanned the hospital records of DM patients with DM from the pre-pandemic period (June 2019 to January 2020) and the post-pandemic period (June 2020 to January 2021) to collect information. We evaluated all patients over 18 with type 2 DM (n: 7,321) who applied to the endocrinology outpatient clinic of Harran University Hospital, and we noted their glycated hemoglobin (A1c) levels, age, and gender. Of these 7,321 cases, 4,501 were pre-pandemic, and 2,820 were post-pandemic. None of them were excluded from this study. We also compared pre and post-pandemic cases according to in a month. Harran University's ethics committee approved the study protocol on November 9, 2020 (protocol number: 19/25) according to the ethical principles for human research specified in the Declaration of Helsinki.

We analyzed the data collected for the study analyzed using SPSS version 22 (SPSS Inc., Chicago, Illinois, USA). The data were shown as a mean ± SD. Continuous variables were analyzed using a t-test. We used the X^2^ test to compare categorical data with an A value of p < 0.05 considered to be statistically significant.

## RESULTS

The number of patients who applied to the endocrinology outpatient clinic was significantly lower after the pandemic than in the pre-pandemic period (4,501 *vs* . 2,820; p < 0.001). The mean age of patients was statistically higher (p < 0.001) in the post-pandemic period (51.5 ± 14.0 years) as compared to the pre-pandemic period (49.7 ± 14.5 years). The mean A1c value was significantly higher (p < 0.001) in the post-pandemic period (7.9% ± 2.4%) than in the pre-pandemic period (7.3% ± 1.7%). The proportion of women in both periods was comparable (59.9% for pre-pandemic, 58.6% for post-pandemic; p = 0.304).

**Figure 1:**
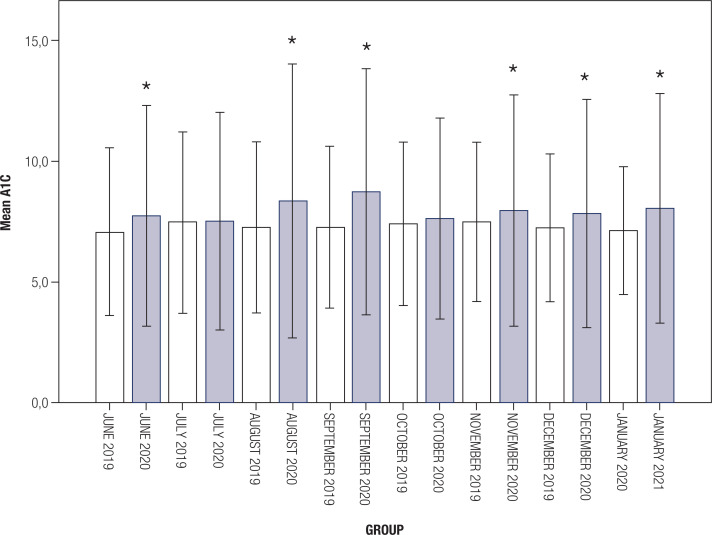
Mean A1c levels of patients during each month.

**Figure 2:**
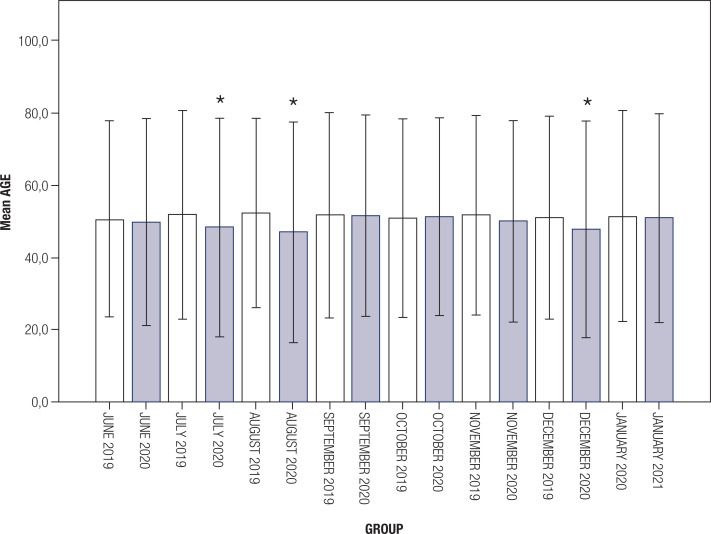
Mean age of patients visiting each month.

In addition, we compared data from pre and post-pandemic periods on a monthly basis. The mean A1c levels in the post-pandemic period were higher compared with the pre-pandemic period in all months, except July and October (p = 0.001 for November, p < 0.001 for others; Figure 1 ). Patients visiting post-pandemic were significantly younger than those visiting pre-pandemic for July, August, and December (p = 0.001 for July, p < 0.001 for August, p < 0.001 for December; Figure 2 ). There were no differences in terms of gender between the groups except in January (p = 0.02). The comparisons for each month are shown in Table 1 .

P values were obtained through Student-t-test. The data are showen as mean ± SD.

P values were obtained through Student-t-test. The data are showen as mean ± SD.

**Table 1:** Month-wise comparison of gender, age and A1c levels of patients

Month	Year	Gender (female %)	Age (years)	A1c (%)
June	2019	61.2	49.4 ± 13.6	7.1 ± 1.7
	2020	58.6	49.8 ± 14.3	7.7 ± 2.3
p		0.435	0.359	<0.001
July	2019	63.1	51.9 ± 14.4	7.5 ± 1.9
	2020	62.2	48.3 ± 15.1	7.5 ± 2.2
p		0.821	0.001	0.642
August	2019	58.8	52.3 ± 13.2	7.3 ± 1.8
	2020	65.6	47.1 ± 15.3	8.3 ± 2.8
p		0.072	<0.001	<0.001
September	2019	61.8	51.8 ± 14.2	7.3 ± 1.7
	2020	55.6	51.6 ± 13.9	8.7 ± 2.5
p		0.072	0.796	<0.001
October	2019	60	50.9 ± 13.7	7.4 ± 1.7
	2020	59.5	51.3 ± 13.6	7.6 ± 2.1
p		0.892	0.684	0.078
November	2019	56.9	51.9 ± 13.8	7.5 ± 1.7
	2020	59.2	50.1 ± 13.9	7.9 ± 2.4
p		0.558	0.076	0.001
December	2019	56.5	51.1 ± 14.1	7.2 ± 1.5
	2020	57.4	47.7 ± 15.0	7.9 ± 2.4
p		0.789	<0.001	<0.001
January	2020	60.6	51.5 ± 14.6	7.1 ± 1.3
	2021	53.1	51.0 ± 14.4	8.0 ± 2.4
p		0.02	0.601	<0.001

## DISCUSSION

In our study on the effects of the pandemic, we found the following: (I) The number of patients admitted to the endocrinology outpatient clinic due to type 2 DM decreased significantly post-pandemic; (II) the number of admissions to the outpatient clinic decreased among elderly patients during the pandemic period; (III) the mean A1c level increased as a result of the pandemic; and (IV) the detrimental effect of the lockdown on metabolic regulation in patients continued for months afterward.

Although the COVID-19 pandemic is global and affects all populations, it had more adverse consequences for patients with chronic illnesses, including diabetes ( 8 ). Routine health care for patients with chronic diseases suffered delays due to the fear of exposure to the coronavirus ( 7 , 8 ). The nationwide lockdowns by governments to prevent its spread have drastically affected the patients with DM, leading to poor glycemic control as they lost their required comprehensive care ( 9 ). Although it is well known that patients with DM have a significantly higher risk of disease severity and associated mortality during SARS-CoV-2 infection, the impact of the COVID-19 pandemic on overall DM control is still largely unclear ( 10 ).

In one study, Ikesu and cols. found that the number of tests for A1c significantly decreased in Japanese acute care hospitals during weeks 9-17 of 2020, compared to weeks 2-8 of 2020 ( 11 ). However, they considered only the number of tests and not the result values obtained from the tests. Xue and cols. showed that the fasting plasma glucose levels in elderly patients with type 2 DM increased during the COVID-19 outbreak ( 12 ). In our study, we observed that A1c levels increased as a result of the pandemic.

Various factors can influence metabolic glucose control during the pandemic in patients with DM. Disasters such as wars, floods, earthquakes, and pandemics tend to affect the patient but also the patient's relatives, followed by the whole social environment ( 13 – 17 ). These situations lead to fear and anxiety in the population, disrupting the natural flow of life ( 13 – 18 ). A pandemic will aggravate negative emotions such as depression and anxiety, which are already prevalent in people with diabetes ( 19 , 20 ). Furthermore, it has been seen that disrupted social communication leading to isolation and loneliness can cause anxiety and depression in elders and negatively affect their health in many ways ( 21 , 22 ). These unhealthy emotions and social isolation can affect glycemic control in people with DM ( 9 , 21 , 23 ). A second factor contributing to aggravated diabetes is the limiting and delaying of outpatient visits for routine management of chronic illnesses to reduce the burden on hospitals and the risk of infection ( 24 ). Third, many people decreased their levels of physical activity because of strict quarantine measures and movement restrictions. The closure of gymnasiums, swimming pools, and exercise clubs, as well as restricted access to open spaces and free movement, reduced the opportunity for exercise ( 23 ). It has been found that increased insulin resistance and worsening glucose control are significantly associated with uninterrupted sitting for prolonged periods ( 25 ). Fourth, quarantine has affected eating habits in different ways from society to society, leading to some negative consequences ( 26 ). Although homemade meals were preferred during the pandemic over fast foods, the unrestricted consumption of oily and high-calorie homemade food such as pastries, French fries, nuts, and dried fruits increased ( 27 – 29 ). At the same time, the frequency of meals also increased due to quarantine and stress ( 27 ). The tendency to gain weight increased, especially in individuals who were already overweight, obese, or older ( 27 - 30 ). Fifth, the frequency of smoking increased significantly among smokers during the quarantine ( 31 , 32 ). The link between smoking and poor metabolic control of diabetes is already well established ( 33 ). Lastly, Kostoglou-Athanassiou and cols. have already shown that vitamin D deficiency contributes to poor glycemic control of diabetes ( 34 ). Home isolation during the pandemic caused a drastic reduction in the hours spent outside in sunlight, leading to the prevalence of low vitamin D levels ( 34 ).

On March 22, 2020, in Turkey, individuals over the age of 65 and those suffering from chronic diseases were restricted from leaving their homes within the scope of pandemic measures. Since the spread of the disease could not be controlled effectively even after these preliminary restrictions, on April 10, the government imposed the first curfew restriction. All citizens were asked to stay at home, except under certain essential circumstances. In June 2020, the measures were relaxed. However, due to the continuing increase in the number of cases, the government reinstated hard restrictions in November 2020. The fact that deterioration of glucose regulation in patients with DM coincided with the lockdown while improvement in glucose regulation coincided with the relaxation of lockdown suggests that lockdowns have a detrimental effect on diabetes control.

As this is study is a retrospective study based on hospital records, data on a diet, physical activity, income, number of births, household members, time of diabetes, and previous insulin use could not be presented. This is an important limitation in our study.

In conclusion, these data suggest that patients with DM need close monitoring during quarantine with several methods such as home care or tele-healthcare. Appropriate diet and exercise programs should also be organized for them to follow at home. It is also essential to provide them with ample social and psychological support to reduce the negative emotions of fear and anxiety arising from social isolation.
